# microRNA-142-3p inhibits apoptosis and inflammation induced by bleomycin through down-regulation of Cox-2 in MLE-12 cells

**DOI:** 10.1590/1414-431X20175974

**Published:** 2017-07-03

**Authors:** F. Guo, S.C. Lin, M.S. Zhao, B. Yu, X.Y. Li, Q. Gao, D.J. Lin

**Affiliations:** 1 Department of Respiratory Medicine, Shandong Provincial Hospital Affiliated to Shandong University, Jinan, Shandong, China; 2 Department of Respiratory Medicine, Yantai Affiliated Hospital of Binzhou Medical University, Yantai, Shandong, China; 3 Department of Respiratory Medicine, Binzhou Medical University Hospital, Binzhou, Shandong, China

**Keywords:** miR-142-3p, MLE-12 cells, Bleomycin, Apoptosis, Pro-inflammatory cytokines, Idiopathic pulmonary fibrosis

## Abstract

microRNA (miR)-142-3p is implicated in malignancy and has been identified as a biomarker for aggressive and recurrent lung adenocarcinomas. This study aimed to evaluate the inhibitory effect of miR-142-3p on apoptosis and inflammation induced by bleomycin in MLE-12 cells. MLE-12 cells were first transfected either with miR-142-3p mimic or miR-142-3p inhibitor and then the cells were exposed to 50 μg/mL of bleomycin. Thereafter, cell viability, apoptosis and the expression of pro-inflammatory cytokines were assessed using CCK-8, flow cytometry, RT-PCR and western blot analyses. Cox-2, PI3K, AKT and mTOR expressions were detected by western blotting after bleomycin was administered together with NS-398 (an inhibitor of Cox-2). As a result, cell viability was significantly decreased, as well as apoptosis and the expression of IL-1 and TNF-α were remarkably increased after 50 and 100 μg/mL of bleomycin administration. miR-142-3p overexpression alleviated bleomycin-induced apoptosis and overproduction of these two pro-inflammatory cytokines, while miR-142-3p suppression exhibited completely opposite results. Up-regulation of Cox-2 and inactivation of PI3K/AKT/mTOR were found in bleomycin-pretreated cells, while these abnormal regulations were partially abolished by miR-142-3p overexpression and NS-398. In conclusion, this study demonstrated that miR-142-3p overexpression protected bleomycin-induced injury in lung epithelial MLE-12 cells, possibly via regulating Cox-2 expression and PI3K/AKT/mTOR signaling pathway. These findings provide evidence that miR-142-3p may be a therapeutic strategy for idiopathic pulmonary fibrosis (IPF) treatment.

## Introduction

MicroRNAs (miRNAs) are approximately 22 nucleotides long non-coding RNA molecules that have been identified as important regulators in gene expression post-transcription. Approximately one-third of human genes are regulated and targeted by miRNAs (1,2). Previous studies have reported that miRNAs are associated with a number of diseases, including cancers, neurodegenerative diseases, diabetes, and inflammation. miR-142-3p is one of the miRNAs, and recent literature has reported its functional effects on various cell lines and diseases. For instance, Lei et al. (3) demonstrated that miR-142-3p influenced the proliferation of non-small cell lung cancer cells through repression of TGFβR1. miR-142-3p have been highlighted as a key molecular player in IL-1β-mediated synaptic dysfunction, which indicated its neuroprotective role in multiple sclerosis (4).

It is well known that idiopathic pulmonary fibrosis (IPF) is a chronic and progressive form of interstitial lung disease characterized by an intricate cytokine network and abnormal deposition of mesenchymal cells (5). Inflammation is triggered by infection or tissue injury involving coordinated recruitment of blood components (plasma and leukocytes) at the site of infection or injury (6,7). These initial events are followed by production of various inflammatory mediators, which include prostaglandins, leukotrienes, prostacyclins, lymphokines, interferon-α (IFN-α), IFN-γ, interleukin (IL)-1, IL-8, histamine, 5-hydroxytryptamine, tumor necrosis factor-α (TNF-α), vasoactive amines, eicosanoids and products of proteolytic cascades (8). Currently, treatment modalities available for IPF are ineffective at halting the disease progression. It is a well-established fact that regulation of gene transcription in health and disease involves several non-redundant mechanisms. Alveolar epithelial cell death has been hypothesized as an initiating mechanism underlying bleomycin-induced lung injury and fibrosis.

Cyclooxygenase (Cox)-2 is a cytokine-inducible enzyme that is present in the nuclear membrane and luminal side of the endoplasmic reticulum (9). It has been reported as a pivotal factor in multiple physiological functions and pathological processes, including inflammation and tumorigenesis (10). Previous studies have shown that Cox-2 has a negative correlation with the expression of several miRNAs, such as miR-26b (11), miR-203 (12), and miR-335 (13).

In the present study, bleomycin was used to injure lung epithelial MLE-12 cells. miR-142-3p expression in cells was altered by transfection with miR-142-3p mimic or miR-142-3p inhibitor, and the changes in cell viability, apoptosis, and secretions of pro-inflammatory factors were detected to assess the impact of miR-142-3p expression on bleomycin-induced injury. In addition, correlations between miR-142-3p and Cox-2 as well as PI3K/AKT/mTOR pathways were detected to reveal the underling molecular mechanism in which miR-142-3p protected MLE-12 cells from bleomycin.

## Material and Methods

### Cell culture and bleomycin treatment

MLE-12 mouse lung epithelial type II cells were purchased from the American Type Culture Collection (USA). Cells were cultured in HITES (hydrocortisone, insulin, transferrin, estrogen) medium (RPMI1640, 2% FBS, 5 mg/mL insulin, 10 mg/mL transferrin, 30 nM sodium selenite, 10 nM hydrocortisone, 10 nM b-estradiol, 10 nM HEPES). MLE-12 cells were treated with bleomycin from 10 to 100 μg/mL for 24 h. NS-398 (20 μM) was used as a Cox-2 inhibitor.

### CCK-8 assay

MLE-12 cells were seeded on 96-well plate with 5000 cells/well. Cell viability was assessed by the cell counting kit-8 (CCK-8, Dojindo Molecular Technologies, Japan). Briefly, after stimulation, the CCK-8 solution was added to the culture medium, and the cultures were incubated for 1 h at 37°C in humidified 95% air and 5% CO_2_. The absorbance was measured at 450 nm using a microplate reader (Bio-Rad, USA).

### Apoptosis assay

Cell apoptosis analysis was performed using propidium iodide (PI) and fluorescein isothiocyanate (FITC)-conjugated Annexin V staining. Briefly, cells were washed in phosphate buffer saline (PBS) and fixed in 70% ethanol. Fixed cells were then washed twice in PBS and stained in PI/FITC-Annexin V in the presence of 50 μg/mL RNase A (Sigma-Aldrich, USA), and then incubated for 1 h at room temperature in the dark. Flow cytometry analysis was done by using a FAC scan (Beckman Coulter, USA). The data were analyzed by using FlowJo software (Tree Star, USA).

### Cell transfection

Mature miR-142-3p mimic, miR-142-3p inhibitor and their corresponding controls, i.e., mimic control and inhibitor control, were designed and synthesized by GenePharma (China). The cell transfection was performed using lipofectamine 3000 (Invitrogen, USA) according to the manufacturer's instructions.

### Quantitative real time PCR

Total RNA was extracted with TRIzol reagent (Sigma-Aldrich) according to the manufacturer's protocol and 2 µg were reverse-transcribed with the Omniscript RT kit (Qiagen, Italy) using random primers (1 mM) at 37°C for 1 h. Real time PCR was performed in triplicate in 20 mL reaction volumes using the Power SYBER Green PCR Master Mix (Applied Biosystems, USA). All primers were purchased from Invitrogen. Real time PCR reactions were carried out in a MJ Mini™ Personal Thermal Cycler apparatus (Bio-Rad Laboratories, USA). Melting curves were obtained by increasing the temperature from 60 to 95°C with a temperature transition rate of 0.5°C/s. The comparative threshold cycle number (C_T_) method was used to assess the relative quantification of gene expression. The fold change of the target gene was calculated as 2^-ΔΔCT^. The internal controls were GAPDH for IL-1, TNF-α, Cox2, and U6 for miR-142-3p.

### Western blot

The protein used for western blotting was extracted using RIPA lysis buffer (Beyotime Biotechnology, China) supplemented with protease inhibitors (Roche). The proteins were quantified using the BCA™ Protein Assay Kit (Pierce, USA). The western blot system was established using a Bio-Rad Bis-Tris gel system according to the manufacturer's instructions. Primary antibodies were prepared in 5% blocking buffer at a dilution of 1:1000, and were then incubated with the membrane at 4°C overnight, followed by wash and incubation with secondary antibody marked by horseradish peroxidase for 1 h at room temperature. After rinsing, the polyvinylidene difluoride (PVDF; Millipore, USA) membrane carrying blots and antibodies was transferred into the Bio-Rad ChemiDoc™ XRS system, and then 200 μL Immobilon western chemiluminescent HRP substrate (Millipore) was added to cover the membrane surface. The signals were captured and the intensity of the bands was quantified using Image Lab™ Software (Bio-Rad).

### Statistical analysis

All experiments were repeated three times. The results of multiple experiments are reported as means±SD. Statistical analyses were performed using SPSS 19.0 (IBM, USA) statistical software. The P values were calculated using a one-way analysis of variance (ANOVA) and P<0.05 was considered to be statistically significant.

## Results

### Bleomycin induced cell injury and up-regulation of pro-inflammatory factors in MLE-12 cells

**Figure 1.**
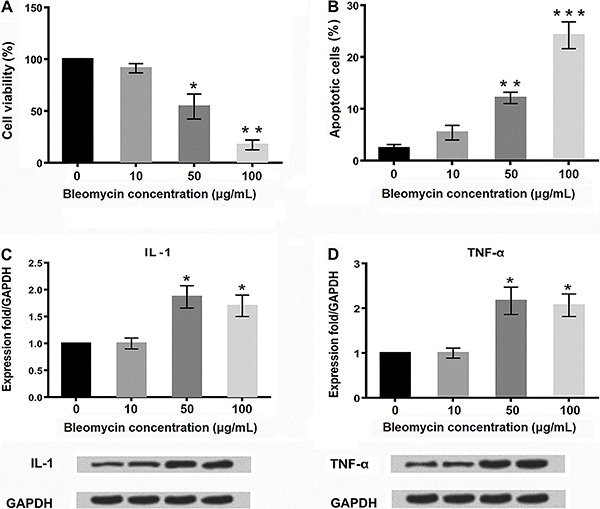
Bleomycin-induced cell injury and up-regulation of pro-inflammatory factors in MLE-12 cells. A series of different concentrations (0, 10, 50, and 100 μg/mL) of bleomycin were applied to MLE-12 cells, and then cell viability (*A*), apoptotic cells rate (*B*), expression level of IL-1 (*C*), and expression level of TNF-α (*D*) were assessed by CCK-8, flow cytometry, and western blot analyses, respectively. Data are reported as means±SD. *P<0.05, **P<0.01, ***P<0.001 compared to the control group (0 μg/mL bleomycin) (ANOVA).

Cell viability decreased significantly (less than 50%) when MLE-12 cells were treated with bleomycin at the concentrations of 50 and 100 µg/mL compared to the control group (P<0.05 or P<0.01; Figure 1A). At the same concentration of bleomycin, apoptosis was found to be significantly higher (more than15%) compared to the control group (P<0.01 or P<0.001; Figure 1B).

The expression of pro-inflammatory cytokines, i.e., IL-1 and TNF-α, were assessed. Significance increases in the expression of these two factors were found after MLE-12 cells were treated with bleomycin. Expression of IL-1 increased significantly by 1.5-fold, at both the doses of 50 and 100 µg/mL of bleomycin compared to the control group (P<0.05; Figure 1C). Similarly, expression of TNF-α also increased significantly by 2-fold at both the doses of 50 and 100 µg/mL of bleomycin compared to the control group (P<0.05; Figure 1D).

### Effect of transfection on miR-132-4p expression in MLE-12 cells

**Figure 2.**
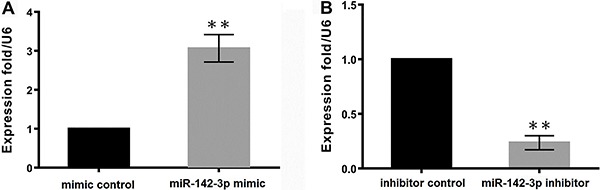
Effect of transfection on miR-132-4p expression in MLE-12 cells. MLE-12 cells were transfected with either miR-142-3p mimic (*A*) or miR-142-3p inhibitor (*B*), and the expression of miR-132-4p in cells was then detected by RT-PCR. Data are reported as means±SD. **P<0.01 (ANOVA).

As shown in Figure 2A and B, after miR-142-3p mimic and miR-142-3p inhibitor transfection, their expressions were more than 3-fold and less than 0.5-fold, respectively, compared to their corresponding controls. These results indicated that miR-142-3p was successfully overexpressed and suppressed in MLE-12 cells after transfection.

### Effect of miR-142-3p overexpression on injured MLE-12 cells and pro-inflammatory cytokines secretion

**Figure 3.**
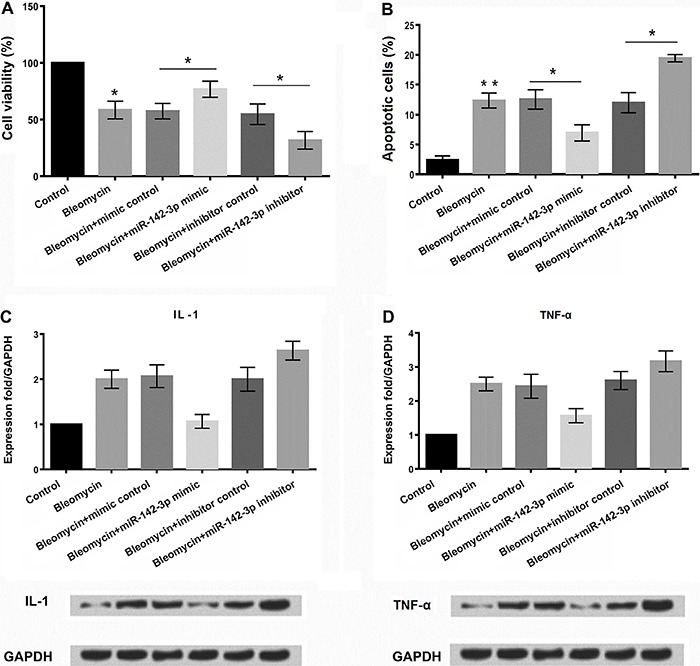
Effect of miR-142-3p overexpression on MLE-12 cells injury and pro-inflammatory cytokines secretion. MLE-12 cells were first transfected either with miR-142-3p mimic or miR-142-3p inhibitor and the cells were then exposed to 50 μg/mL of bleomycin. Thereafter, cell viability (*A*), apoptosis (*B*), and the expression of IL-1 (*C*) and TNF-α (*D*) were, respectively, assessed by using CCK-8, flow cytometry, RT-PCR and western blot analyses. Data are reported as means±SD. *P<0.05, **P<0.01 compared to their corresponding controls (ANOVA).

miR-142-3p overexpression significantly alleviated bleomycin-induced cell viability reduction and apoptotic cells increase (both P<0.05; Figure 3A and B). In addition, miR-142-3p overexpression alleviated both IL-1 and TNF-α overproductions induced by bleomycin (Figure 3C and D). Conversely, miR-142-3p suppression affected MLE-12 cells viability, apoptosis, and IL-1 and TNF-α levels in a completely opposite manner.

### Effect of miR-142-3p overexpression on Cox-2 expression on MLE-12 cells challenged by bleomycin

**Figure 4.**
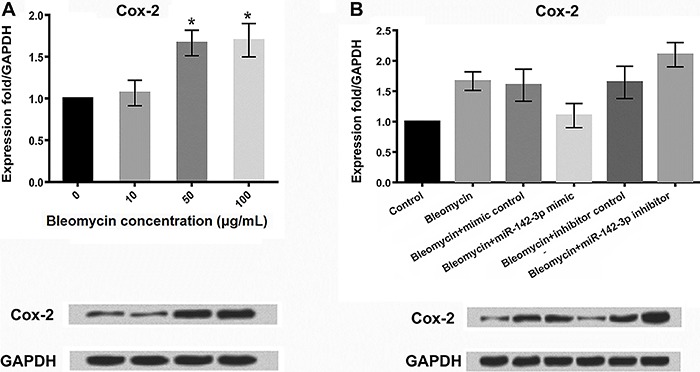
Effect of miR-142-3p overexpression on Cox-2 expression, when challenged by bleomycin in MLE-12 cells. *A*, Different concentrations (0, 10, 50, and 100 μg/mL) of bleomycin were applied in MLE-12 cells, and the expression of Cox-2 was detected. *B*, Cells were first transfected either with miR-142-3p mimic or miR-142-3p inhibitor and the cells were then exposed to 50 μg/mL of bleomycin; thereafter, Cox-2 expression level was determined. Data are reported as means±SD. *P<0.05 compared to the control group (0 μg/mL of bleomycin; ANOVA).

Cox-2 expression was found to be up-regulated by 1.5-fold after bleomycin administration at both 50 and 100 µg/mL doses in MLE-12 cells (Figure 4A). miR-142-3p overexpression could decrease Cox-2 expression induced by bleomycin (50 µg/mL) back to the 1-fold/GAPDH level (Figure 4B). However, miR-142-3p suppression could slightly promote the regulation of bleomycin on Cox-2 expression.

### Effect of miR-142-3p on Cox-2 and PI3K/AKT/mTOR pathway in MLE-12 cells

**Figure 5.**
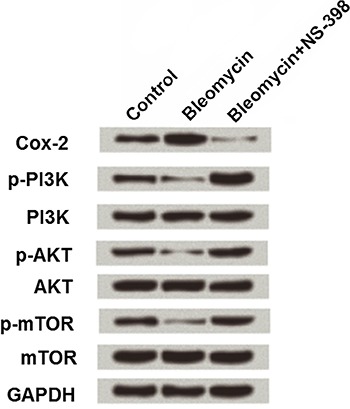
Effect of bleomycin on Cox-2 and PI3K/AKT/mTOR pathway in MLE-12 cells. MLE-12 cells were subjected to 50 μg/mL of bleomycin alone or together with 20 μM NS-398, and Cox-2, PI3K, AKT, and mTOR expressions were detected.

MLE-12 cells were pretreated with 50 µg/mL bleomycin alone or together with 20 μM NS-398 (a Cox-2 inhibitor), and then the expression changes in Cox-2, PI3K, AKT and mTOR were detected by western blotting. As shown in Figure 5, 50 µg/mL bleomycin up-regulated Cox-2 by 1.5-fold compared to the expression in the control group (1-fold), while Cox-2 was decreased to 0.2-fold in cells treated with both bleomycin and NS-398. Besides, down-regulations of p-PI3K (0.7-fold), p-AKT (0.5-fold), and p-mTOR (0.5-fold) were observed in bleomycin treated cells, while these three factors were highly expressed in cells treated with bleomycin and NS-398, with 1.7-, 1.5-, and 1.2-fold, respectively.

**Figure 6.**
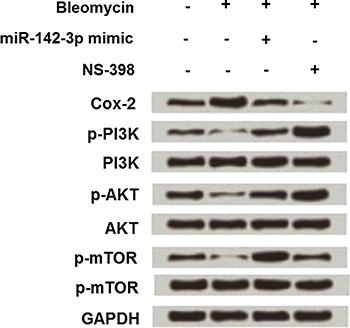
Effect of miR-142-3p overexpression on Cox-2 and PI3K/AKT/mTOR pathway in MLE-12 cells. miR-142-3p overexpressing-cells were subjected to 50 μg/mL of bleomycin alone or together with 20 μM NS-398, and Cox-2, PI3K, AKT, and mTOR expressions were detected.

miR-142-3p overexpressing cells were then treated with 50 µg/mL bleomycin alone or together with NS-398, and the expression changes of Cox-2, PI3K, AKT and mTOR were assessed again. As shown in Figure 6, miR-142-3p overexpression notably reduced bleomycin-induced Cox-2 up-regulation (1.7- to 0.9-fold), as well as PI3K (0.3- to 1.2-fold), AKT (0.4- to 1.1-fold) and mTOR (0.2- to 2-fold) activation.

## Discussion

In the present study, 50 µg/mL of bleomycin was applied into MLE-12 cells to mimic an *in vitro* IPF model. After bleomycin administration, cell viability was reduced, apoptotic cell number was increased and two pro-inflammatory factors (IL-1 and TNF-α) were over-produced, which indicated MLE-12 cells were injured by bleomycin. Following miR-142-3p mimic transfection, cell injury and pro-inflammatory factors production were alleviated, indicating the protective role of miR-142-3p in bleomycin-induced injury. The expression of Cox-2 was up-regulated by a high dose of bleomycin, while miR-142-3p overexpression partially abolished the regulatory impacts of bleomycin on Cox-2. Besides, NS-398 was applied together with bleomycin, and we found that miR-142-3p negatively regulated Cox-2 via PI3K/AKT/mTOR signaling pathway.

Over the last decade, miR-142-3p has been emerging as a major regulator of many biological processes, including cell apoptosis and inflammatory response. For instance, miR-142-3p inhibited hypoxia/reoxygenation-induced apoptosis of cardiomyocytes (14) and participated in the regulation of aging-related immune responses (15). In the lung, miR-142-3p is involved in malignancy and has been reported to be an early biomarker for aggressive and recurrent lung adenocarcinomas (16). Furthermore, Carraro et al. (17) demonstrated that miR-142-3p balanced proliferation and differentiation of mesenchymal cells during lung development, which indicated a potential role of miR-142-3p in lung diseases such as IPF in which cellular proliferation and differentiation of mesenchymal cells is unbalanced. In the present study, miR-142-3p overexpression exerted protective roles in bleomycin-induced injury in MLE-12 cells, and cell apoptosis and production of pro-inflammatory factors were inhibited after miR-142-3p mimic transfection. These findings further confirmed the hypothesis proposed by Carraro et al. (17), that miR-142-3p might be a potential therapeutic alternative for IPF.

Cox-2 is a critically important mediator of apoptosis and inflammation that significantly influences tumor cells fate (18,19). For example, Cox-2 induction and subsequent Cox-2-dependent activation of PPARγ as a mechanism by which lovastatin lactone induced human lung cancer cell death (20). In the normal lung, Cox-2 has been also reported as a pivotal factor in the activation of the apoptotic and inflammatory pathway (21). In the current study, Cox-2 was up-regulated after bleomycin administration, which indicated that apoptosis and inflammation of MLE-12 cells were induced via regulating Cox-2. Additionally, miR-142-3p overexpression could suppress Cox-2 expression, which provided the first evidence that miR-142-3p protected bleomycin-induced injury via negative regulation of Cox-2.

PI3K/AKT/mTOR is a major pathway mediating cell survival by promoting cell proliferation and inhibiting apoptosis (22,23) and is emerging as a potential therapeutic target for IPF (24). Previous studies have also reported the correlations between Cox-2 expression and PI3K/AKT/mTOR signaling pathway (25). A study in human renal mesangial cells suggested that PI3K/AKT activation participates in TGF-β-mediated induction of Cox-2 protein expression (26). The findings in this study were in line with these previous studies that the up-regulation of Cox-2 induced by bleomycin had a negative correlation with the activation of PI3K, AKT and mTOR. Besides, miR-142-3p overexpression could alleviate bleomycin-induced up-regulation of Cox-2 and the inactivation of PI3K/AKT/mTOR. Moreover, NS-398 was used in this study to confirm the regulatory role of Cox-2 in PI3K/AKT/mTOR signaling pathway. Administration of NS-398 in the lung alleviated bleomycin-induced fibrosis in mice by inducing apoptosis and inflammation (27). Thus, we inferred that miR-142-3p protected bleomycin-induced injury in MLE-12 cells via regulating Cox-2 and PI3K/AKT/mTOR signaling pathway.

In conclusion, this study demonstrated that miR-142-3p overexpression protected bleomycin-induced injury in lung epithelial MLE-12 cells possibly via regulating Cox-2 expression and PI3K/AKT/mTOR signaling pathway. These findings provide evidence that miR-142-3p may be a therapeutic strategy for IPF treatment. Further investigations are still needed to reveal the exact underlying molecular mechanisms in which miR-142-3p functions in lung epithelial cells.
